# Dealing with Dementia: Insights from the Latest German Guidelines

**DOI:** 10.1192/j.eurpsy.2025.159

**Published:** 2025-08-26

**Authors:** J. Benninghoff

**Affiliations:** Zentrum für Altersmedizin und Entwicklungsstörungen - ZfAE, kbo, München, Germany

## Abstract

**Abstract:**

The management of Behavioral and Psychological Symptoms of Dementia (BPSD) remains a key challenge in clinical practice. This presentation will provide an overview of the latest German guidelines, highlighting evidence-based recommendations for pharmacological and non-pharmacological interventions. Key updates emphasize a patient-centered approach, focusing on prevention, risk assessment, and the integration of innovative care strategies. By examining these guideline revisions, the session aims to equip healthcare professionals with practical insights to optimize dementia care and improve patient outcomes in line with current best practices in Germany.

**Disclosure of Interest:**

None Declared

